# Corrigendum to “Rare but Not so Rare? The Evolving Spectrum of Whipple's Disease”

**DOI:** 10.1155/2024/9809135

**Published:** 2024-10-11

**Authors:** John M. Conly, B. Lynn Johnston

**Affiliations:** ^1^Foothills Medical Centre, University of Calgary and Alberta Health Services, Calgary, Alberta, Canada; ^2^Retired, Halifax, Nova Scotia, Canada

In the article titled “Rare but Not so Rare? The Evolving Spectrum of Whipple's Disease” [1], an article used as a source was inadvertently omitted from the references list, for which some overlapping text exists [2]. The authors wish to express their sincere regret for this oversight whilst preparing the article.
